# The international, prospective COSMOS (CytOSorb® TreatMent Of Critically Ill PatientS) Registry: results from the first 300 patients

**DOI:** 10.1186/s44158-026-00362-2

**Published:** 2026-02-25

**Authors:** Ricard Ferrer, Matthias Thielmann, Moritz Unglaube, Thomas Kirschning, Andreas Baumann, Julian Kreutz, Andreas Kribben, Bartosz Tyczynski, Ulf Guenther, Dietrich Henzler, Christina Scharf, Nuno Germano, Martin Bellgardt, Aschraf El-Essawi, Philipp Hohlstein, Thomas Guenther, P. Christian Schulze, Filippo Aucella, Mario Marquez Fernandez, Markus Koestenberger, Gabriella Bottari, Jorge Hidalgo, Jean-Louis Teboul, Dana Tomescu, Teresa Klaus, Weihong Fan, Joerg Scheier, Efthymios N. Deliargyris, Fabio Silvio Taccone

**Affiliations:** 1 Intensive Care Department, Vall d’Hebron University Hospital, Shock, Organ Dysfunction and Resuscitation Research Group (SODIR), Barcelona, Spain; 2 Department of Thoracic and Cardiovascular Surgery, West German Heart & Vascular Center Essen, University Hospital Duisburg-Essen, Essen, Germany; 3 Department of Intensive Care, Helios Dr. Horst-Schmidt Klinik Wiesbaden, Wiesbaden, Germany; 4 Department of Cardiothoracic Surgery, Heart and Diabetes Center NRW, Bad Oeynhausen, Germany; 5 Department of Anesthesiology, Intensive Care Medicine and Pain Management, BG University Hospital Bergmannsheil, Medical Faculty of Ruhr University Bochum, Bochum, Germany; 6 Department of Cardiology, Angiology, and Intensive Care Medicine, University Hospital, Philipps University of Marburg, Marburg, Germany; 7 Department of Nephrology, University Duisburg-Essen, University-Hospital Essen, Essen, Germany; 8 Department of Medical Intensive Care, University Clinic Essen, Essen, Germany; 9 Klinikum Oldenburg AoeR, University Clinic of Anesthesiology and Intensive Care, Oldenburg, Germany; 10 Department of Anesthesiology, Surgical Intensive Care, Emergency and Pain Medicine, Ruhr-University Bochum, Klinikum Herford, Herford, Germany; 11 Department of Anesthesiology, LMU, Munich, Germany; 12 Department of Intensive Care, Hospital Curry Cabral, Unidade de Cuidados Intensivos Polivalente, Lisbon, Portugal; 13 Department of Anesthesiology and Intensive Care Medicine, St Josef-Hospital Bochum, University Hospital of the Ruhr-University Bochum, Bochum, Germany; 14 Department of Thoracic and Cardiovascular Surgery, University Medical Center Goettingen, Goettingen, Germany; 15 Department for Gastroenterology, Metabolic Disorders and Intensive Care Medicine (Department of Medicine III), RWTH-University Hospital Aachen, Aachen, Germany; 16 Department of Cardiovascular Surgery, German Heart Center Munich, School of Medicine & Health, TUM University Hospital, Technical University of Munich, Munich, Germany; 17 Department of Internal Medicine and Cardiology, University of Jena, Jena, Germany; 18 Department of Nephrology and Dialysis, “Casa Sollievo Della Sofferenza” Foundation, Scientific Institute for Research and Health Care, San Giovanni Rotondo, FG Italy; 19 Intensive Care Department, Hospital Universitario Juan Ramón Jiménez – HJRJ, Huelva, Spain; 20 Department of Anesthesiology and Intensive Care, Hospital Klagenfurt Am Woerthersee, Klagenfurt Am Woerthersee, Austria; 21 Pediatric Intensive Care Unit, Children Hospital Bambino Gesù, IRCCS, Rome, Italy; 22 General Intensive Care Unit and COVID-19 Unit, Belize Healthcare Partners, Belize City, Belize; 23 Paris-Saclay Medical School, Paris-Saclay University, Le Kremlin-Bicêtre, France; 24 Department of Anesthesiology and Critical Care III, “Carol Davila” University of Medicine and Pharmacy, Fundeni Clinical Institute, Bucharest, Romania; 25 Medical Affairs, CytoSorbents Europe GmbH, Berlin, Germany; 26 Medical Affairs, CytoSorbents Corporation and CytoSorbents Medical Inc., Princeton, NJ USA; 27 Department of Intensive Care, Hôpital Universitaire de Bruxelles (HUB), Université Libre de Bruxelles (ULB), Brussels, Belgium

**Keywords:** CytoSorb, Hemoadsorption, Hemoperfusion, Adsorption, Blood purification, Hyperinflammation, Sepsis, Septic shock, Liver failure, Rhabdomyolysis, Registry

## Abstract

**Introduction:**

Blood purification techniques are being investigated as adjunctive options in critically ill patients not only to treat severe inflammation but also to remove harmful substances such as myoglobin in rhabdomyolysis. Yet, the available evidence is limited, and further research is needed to clarify their clinical benefits.

**Methods:**

The international prospective COSMOS Registry (NCT05146336, 23 Nov 2021) tracks CytoSorb® (CS) utilization patterns and outcomes in critical care settings. Clinical assessment was performed before, during, and after CS treatment, with a 90-day follow-up. Device-related adverse effects were reported by investigators as the safety evaluation. Data were analyzed according to a pre-specified statistical plan using descriptive statistics and paired tests to compare pre- and post-treatment values, with subgroup and safety analyses performed.

**Results:**

A total of 300 adult patients (30.3% female, mean age 59 ± 15 years) from 22 sites were included in this analysis. The most common indications for CS therapy (multiple indications possible per patient) were septic shock (48.3%), rhabdomyolysis (12.8%), cardiogenic shock (11.5%), liver failure (11.5%), and acute respiratory distress syndrome (ARDS; 5.0%). On average, each patient received 3.3 ± 3.3 adsorbers, with 27.9% of patients receiving 4 or more adsorbers. CS was integrated in conjunction with kidney replacement therapy (75.6%), standalone hemoperfusion (7.1%), intermittent hemodialysis (IHD; 10.6%), extracorporeal membrane oxygenation (ECMO; 3.9%), and sustained low-efficiency daily dialysis (SLEDD; 4.9%). At baseline, median (interquartile range, IQR) APACHE II and SOFA scores were 24 [18, 30] and 12 [9, 15], respectively. Fluid balance improved from +1675 [141, 3348] mL pre-CS to +115 [−1100, 1495] mL post-CS, and norepinephrine requirements decreased from 0.21 [0.09, 0.40] µg/kg/min to 0.08 [0.02, 0.22] µg/kg/min (*p* < 0.0001 for both). Ratio of partial pressure of oxygen in arterial blood to the fraction of inspiratory oxygen concentration (P/F ratio) improved from 120 [72, 208] to 176 [115, 255] (*p* < 0.0001). Platelet counts decreased from 123 [76, 185] to 72 [42, 118] × 10^9^/L (*p* < 0.0001), while albumin levels remained stable from 2.6 [2.3, 3.1] to 2.5 [2.3, 3.0] g/dL (*p* = 0.112). ICU mortality was 33.1%, which was lower than mortality estimates historically associated with comparable APACHE II and SOFA scores. No serious adverse effects related to the device or device deficiencies were reported.

**Conclusions:**

Real-world CytoSorb® use as part of standard care in critically ill patients was associated with improvements in several clinical and laboratory parameters; however, these findings should be interpreted cautiously given the observational design and absence of a control group. Observed mortality was lower than mortality estimates historically associated with established severity scores.

## Introduction

CytoSorb® (CS) is a hemoadsorption cartridge consisting of porous polymer beads that can remove a wide range of small and middle-sized hydrophobic substances, including cytokines, toxins, and other harmful substances from whole blood [1]. This mechanism has been particularly beneficial in conditions like sepsis and other inflammatory diseases like acute respiratory distress syndrome (ARDS), where excess cytokines are a major driver of organ dysfunction [2, 3]. Additionally, the ability to also remove bilirubin and bile acids as well as myoglobin expands the spectrum of critical care indications for CS to include acute and acute-on-chronic liver failure (ALF/ACLF), secondary liver dysfunction, or rhabdomyolysis [4, 5]. However, much of the evidence to date comes from small, single-center studies without control groups, leaving gaps in knowledge about the real-world performance of this therapy in diverse patient populations.

While randomized controlled trials are considered the gold standard for evaluating clinical interventions, real-world evidence (RWE) is increasingly recognized for its critical role in understanding the practical applications of therapies outside the controlled environment of clinical trials [6]. RWE provides insights into how therapies perform in routine clinical practice, where patient characteristics, comorbidities, and institutional practices can vary widely. This is especially relevant for CS since it can be used in a wide range of critical care conditions. Furthermore, RWE collection is necessary for post-market surveillance as required by Medical Device Regulations [7].

The COSMOS (**CytoSorb®** Treat**M**ent **O**f Critically Ill Patient**S**) Registry is an international, prospective multicenter data collection platform designed to capture the real-world effectiveness and safety of CS in critically ill patients [8]. This planned interim report from the first 300 adult patients enrolled in the registry aims to provide a comprehensive overview of utilization patterns and clinical performance of CS in the real world. Understanding how CS performs across such varied conditions is crucial for assessing its broader applicability in critical care. In this context, real-world evidence from the COSMOS Registry can help clinicians better understand potential benefits but also potential limitations of CS therapy, refine patient selection criteria, and guide future research directions in the field of hemoadsorption therapy.

## Methods

### Registry study design

The COSMOS Registry is an international, prospective, single-arm observational study conducted in countries where the device is approved for routine clinical use [8]. Sites are selected based on their research credentials and capabilities, routine use of the device, patient enrollment capacity, and interest in participation. Device use should follow applicable current best practice [9–11] and all site personnel receive training to ensure proper use. Investigators are encouraged to review the latest device Instructions for Use for contraindications and precautions. To minimize selection bias, sites are asked to enroll consecutive eligible patients. Institutional Review Board (IRB)/Independent Ethics Committee (IEC) approval was secured at every site and informed consent obtained from all participants before data collection.

### Study population

The current interim analysis focuses on the first 300 adult patients (age ≥ 18 years old) enrolled in the COSMOS Registry from 22 hospitals across Germany, Italy, Spain, Austria, and Portugal. These patients were treated with CS as part of their clinical management for a variety of indications, including septic shock, cardiogenic shock, rhabdomyolysis, and acute liver failure. Among the 300 patients included in this analysis, 268 patients received CytoSorb® and no other adsorption devices. 14 patients were treated with CytoSorb® in combination with other adsorption devices, with a median duration of 2 h for the additional adsorption therapy. For 18 patients, information on the use of other adsorption devices was not available.

Patients were eligible if they were treated with CytoSorb® 300 mL during their ICU stay and if an informed consent for prospective registry participation was obtained by the patient or legally authorized representative. Exclusion criteria included the use of the CytoSorb® for antithrombotic removal only, or intra-operatively during cardiac surgery only.

The registry collects data across a broad range of clinical indications of CS hemoadsorption. These include hyperinflammatory conditions such as septic shock, hemophagocytic lymphohistiocytosis (HLH), cytokine release syndrome (CRS) and CAR-T–related CRS, cardio-pulmonary settings like cardiogenic shock, ARDS, and ECMO/ECLS, and liver or kidney dysfunction including ALF, ACLF, secondary liver dysfunction, and rhabdomyolysis with multiple indications for CS being possible. Further recorded indications are infectious diseases such as COVID-19, influenza, and dengue, as well as post-operative vasoplegic shock, burns, pancreatitis, and drug overdose removal.

### Data collection and study outcomes

Data are systematically collected at defined time points, including screening, 24 h before CS initiation, 24 h after completion, at ICU and hospital discharge, and again at 90-day follow-up. An overview of the baseline characteristics, therapeutic interventions, biomarkers, clinical measurements, and outcome as well as safety variables assessed in this analysis is provided in Table 1. The registry assessed ICU mortality as the primary endpoint, with secondary outcomes including key laboratory, hemodynamic and clinical parameters, longer-term mortality and device safety.

**Table 1:** Baseline characteristics and study outcomes

Domain	Variables/parameters
Screening/baseline characteristics	Demographics: • Age • Sex Comorbid disease/risk scores: • APACHE II score (Acute Physiology and Chronic Health Evaluation) • SOFA score (Sequential Organ Failure Assessment) • Charlson Comorbidity Index
Treatment characteristics	• Indication for CS • Type of extracorporeal circuit used • Need for kidney replacement therapy (KRT) • Number of CS adsorbers used per patient • Duration of CS treatment • Timing of CS therapy initiation relative to ICU admission
Primary outcome parameter	ICU mortality
Secondary outcome parameters	Laboratory biomarkers (pre- versus post-CS): • Interleukin-6 (IL-6) • C-reactive protein (CRP) • Procalcitonin • Lactate • Creatinine • pH • Platelet count • Albumin Clinical parameters (pre- versus post-CS): • Norepinephrine dosage • Fluid balance • Oxygenation (P/F ratio, ratio of partial pressure of oxygen in arterial blood to the fraction of inspiratory oxygen concentration) • Mean arterial pressure (MAP) • MAP/norepinephrine (MAP/NE) index Other outcomes: • 90-day mortality • ICU length of stay (in survivors) • Hospital length of stay Safety parameters: • Serious device-related adverse events • Device deficiencies • Bleeding events • Need for albumin substitution

### Statistical analysis

A pre-specified statistical analysis plan was followed to summarize and analyze the data. For continuous variables, either the median with first and third quartiles [Q1, Q3] or the mean with standard deviation (SD) were reported. For categorical variables, counts and proportions were provided. Baseline data were defined as the time of ICU admission and included demographic information and most commonly used risk scores for critically ill patients. The pre-CS therapy period was defined as the 24 h prior to the start of CS therapy and included assessments for vasopressor use and fluid balance, with the latest available lab and blood gas values. The post-CS therapy period was defined as the 24 h following the end of CS therapy for vasopressor use and fluid balance with the earliest available measurements for lab and blood gas values. Absolute change from the pre-CS period was calculated as the post-CS measurement minus the pre-CS value. The Wilcoxon signed-rank test was used to compare paired pre- and post-CS data to assess changes associated with CS treatment. We applied the Wilcoxon signed-rank test universally because several lab parameters had small sample sizes, non-normal distributions, or extreme outliers, making this test more robust than the paired *t*-test until larger sample sizes reduce these limitations. *p* values less than 0.0001 were reported as <0.0001 in tables; all other *p* values were rounded and displayed using three decimals. Subgroup analyses were performed for clinically relevant categories, e.g., septic shock and KRT use. Kaplan–Meier plots illustrated the probability of survival over time (from the start of CytoSorb up to 90 days) by the subgroups, along with log-rank *p* value. A separate analysis of ICU survivors and non-survivors was also performed. Device safety was evaluated based on investigator-reported adverse events related to the device, recorded until either ICU discharge or death, whichever occurred first. All analyses were performed using SAS 9.4 (SAS, Cary, NC, USA).

## Results

### Patient characteristics

A total of 300 adult patients enrolled at 22 sites in Germany, Spain, Italy, Austria, and Portugal were included in this analysis. Patients’ mean age was 59 ± 15 years, with 30.3% being female (Table 2). Median baseline Acute Physiology and Chronic Health Evaluation (APACHE) II score was 24, with an interquartile range (IQR) of 18 to 30, and median Sequential Organ Failure Assessment (SOFA) score was 12, with IQR of 9 to 15. In the septic shock cohort patients (*n* = 155), APACHE II score was 24 [18, 29] and SOFA 12 [9, 15].

**Table 2:** Baseline characteristics of the first 300 patients in COSMOS Registry (*n* and percent shown for categorial variables, median [Q1, Q3] for continuous variables except age which is presented as mean and standard deviation). Not all patients’ data was available at time of the interim data lock

Baseline characteristics	All patients (*n* = 300)	ICU survivors (*n* = 180)	ICU non-survivors (*n* = 89)
Age (years)			
Mean ± SD	59 ± 15	57 ± 16	64 ± 13
Age group, *n* (%)			
≥65	131/300 (43.7%)	70/180 (38.9%)	45/89 (50.6%)
18–65	169/300 (56.3%)	110/180 (61.1%)	44/89 (49.4%)
Gender, *n* (%)			
Male	209/300 (69.7%)	124/180 (68.9%)	60/89 (67.4%)
Female	91/300 (30.3%)	56/180 (31.1%)	20/89 (32.6%)
Weight (kg)			
*N*	292	176	86
Median [Q1, Q3]	85 [72, 98]	85 [70, 100]	85 [75, 95]
BMI (kg/m^2^)			
*N*	290	176	84
Median [Q1, Q3]	27.2 [24.2, 31.2]	26.8 [24.0, 31.2]	27.4 [24.7, 32.5]
Charlson comorbidity score			
*N*	279	173	75
Median [Q1, Q3]	4 [2, 5]	3 [2, 5]	5 [3, 6]
APACHE II score			
*N*	242	149	85
Median [Q1, Q3]	24 [18, 30]	23 [18, 28]	25 [18, 32]
SOFA score			
*N*	238	156	75
Median [Q1, Q3]	12 [9, 15]	12 [9, 15]	13 [10, 15]

*APACHE* Acute Physiology and Chronic Health Evaluation, *SD* standard deviation, *BMI* body mass index, *SOFA* Sequential Organ Failure Assessment, *Q1* first quartile, *Q3* third quartile

### Treatment modalities and indications of CytoSorb therapy

The therapeutic use of CS in the COSMOS Registry covers a wide spectrum of clinical conditions, with a predominant focus on the treatment of hyperinflammation and organ failure. Also, multiple indications for some patients were possible. Indication for CS included septic shock (*n* = 155, 48.3% of all indications), cardiogenic shock (*n* = 37, 11.5%), rhabdomyolysis (*n* = 41, 12.8%), acute or acute on chronic liver failure (ALF/ACLF) or secondary liver dysfunction (*n* = 37, 11.6%), acute respiratory distress syndrome (ARDS; *n* = 16, 5.0%), and other (*n* = 35, 10.9%). Mean number of adsorbers used per patient was 3.3 ± 3.3, with 28% receiving 4 or more adsorbers. Median duration of a single adsorber was 18 [9.5, 24] hours and total overall duration of treatment was 32 [22.7, 65.8] hours. Information on extracorporeal platforms used for CS integration was available for 283 patients and included continuous renal replacement therapy (*n* = 214, 75.6% of all available info on circuits), standalone hemoperfusion (*n* = 20, 7.1%), intermittent hemodialysis (IHD; *n* = 30, 10.6%), extracorporeal membrane oxygenation (ECMO; *n* = 11, 3.9%), and sustained low-efficiency daily dialysis (SLEDD; *n* = 14, 4.9%).

### Primary and secondary outcomes

**Fig. 1:**
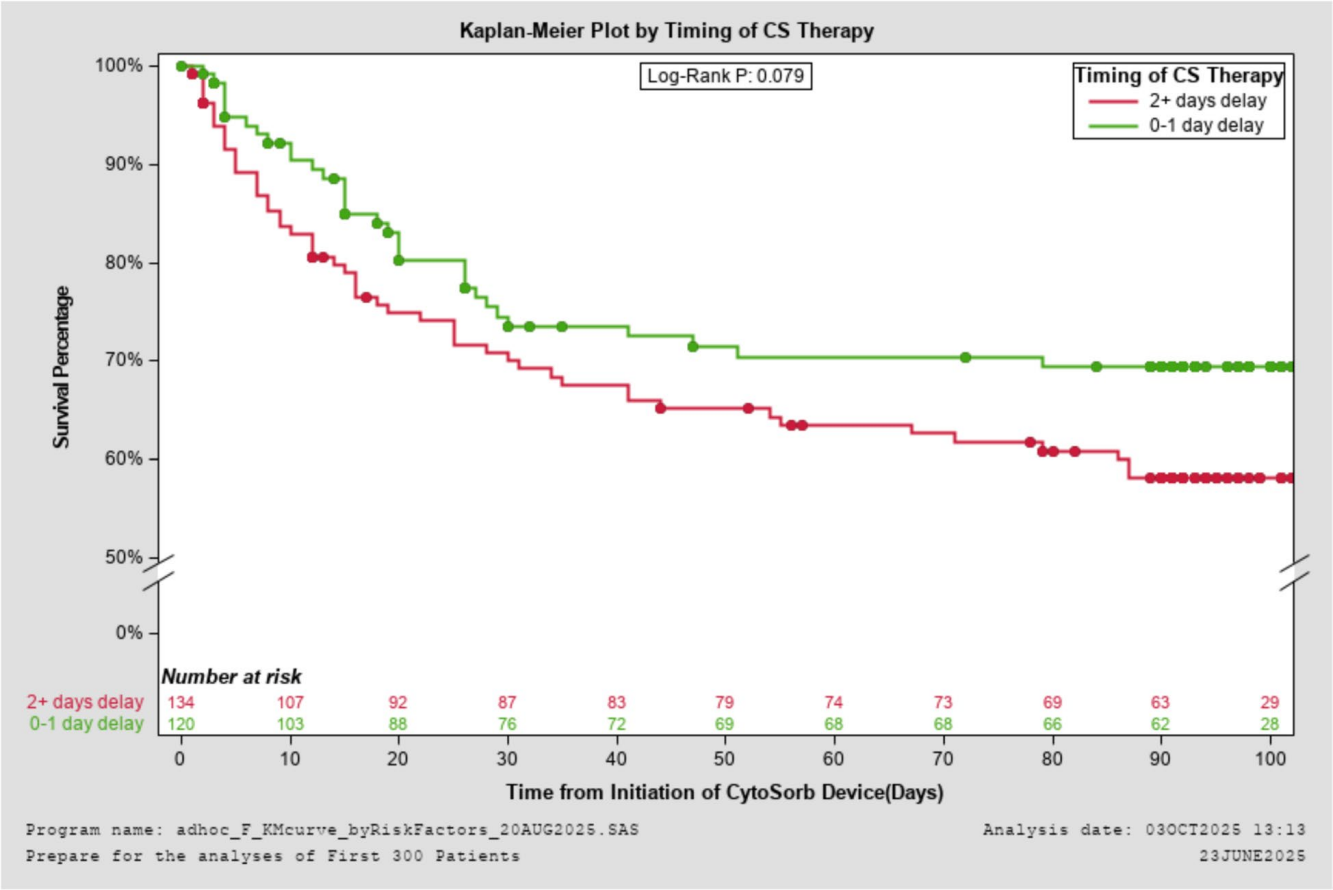
Association of time of CS initiation and survival percentage

The primary outcome of ICU mortality was 33.1% overall and 32.9% in septic shock cohort. Mortality at 90 days was 46.0% for 248 patients with the completion of follow-up. Median length of ICU stay for survivors was 22 [12, 38] days. Table 3 shows an overview of mortality rates in relation to the onset of CS therapy. Survival was numerically higher when CS therapy was initiated within 1 day of ICU admission compared to initiation after more than 1 day (see also Fig. 1). Note that the Kaplan–Meier curve accounts for censored data, unlike incidence rates, which exclude patients lost to follow-up. However, it cannot accurately estimate ICU mortality because patients have varying ICU lengths of stay. Furthermore, patients with immediate CS onset had a shorter ICU stay (16 vs. 27 days, *p* < 0.0001) and hospital stay (30 vs. 42 days, *p* = 0.005).

**Table 3:** Mortality of patients according to onset of CS therapy in relation to ICU admission

	Onset of CS within 0–1 day of ICU admission	Delayed onset (≥2 days after ICU admission)	*p* value
ICU mortality	22.3% (25/112)	29.8% (39/131)	0.189
Hospital mortality	27.3% (30/110)	32.3% (42/130)	0.396
Mortality at 90-day FU	34.7% (35/101)	44.3% (54/122)	0.145

Table 4 summarizes biomarker data pre- versus post-CS use. Of note, in patients where CS was integrated into ECMO or used in hemoperfusion mode without KRT (*n* = 31), creatinine (1.9 [1.1, 3.0] to 1.3 [0.8, 2.0] mg/dL, *p* < 0.0001) and lactate (2.1 [1.2, 2.6] to 1.4 [1.1, 1.6] mmol/L, *p* = 0.037) were also significantly reduced.

**Table 4:** Overview of laboratory findings before versus after CS therapy

Biomarkers	Median levels [IQR] before CS	Median levels [IQR] after CS	*p* value
Interleukin-6 pg/mL, *n* = 58	563 [187, 14,168]	104 [52, 1299]	<0.0001
C-reactive protein mg/dL, *n* = 159	13.0 [3.5, 22.1]	10.8 [4.3, 19.1]	0.082
Procalcitonin ng/mL, *n* = 98	9.4 [2.1, 47.0]	3.3 [1.2, 16.0]	<0.0001
pH, *n* = 233	7.35 [7.30, 7.42]	7.40 [7.36, 7.45]	<0.0001
Lactate mmol/L, *n* = 231	2.4 [1.4, 4.9]	1.4 [1.0, 2.2]	<0.0001
Creatinine mg/dL, *n* = 231	2.1 [1.4, 3.1]	1.4 [0.9, 2.1]	<0.0001
Albumin g/dL, *n* = 122	2.6 [2.3, 3.1]	2.5 [2.3, 3.0]	0.112
Platelets × 10^9^/L, *n* = 237	123 [76, 185]	72 [42, 118]	<0.0001

*CS* CytoSorb, *IQR* interquartile range

**Fig. 2:**
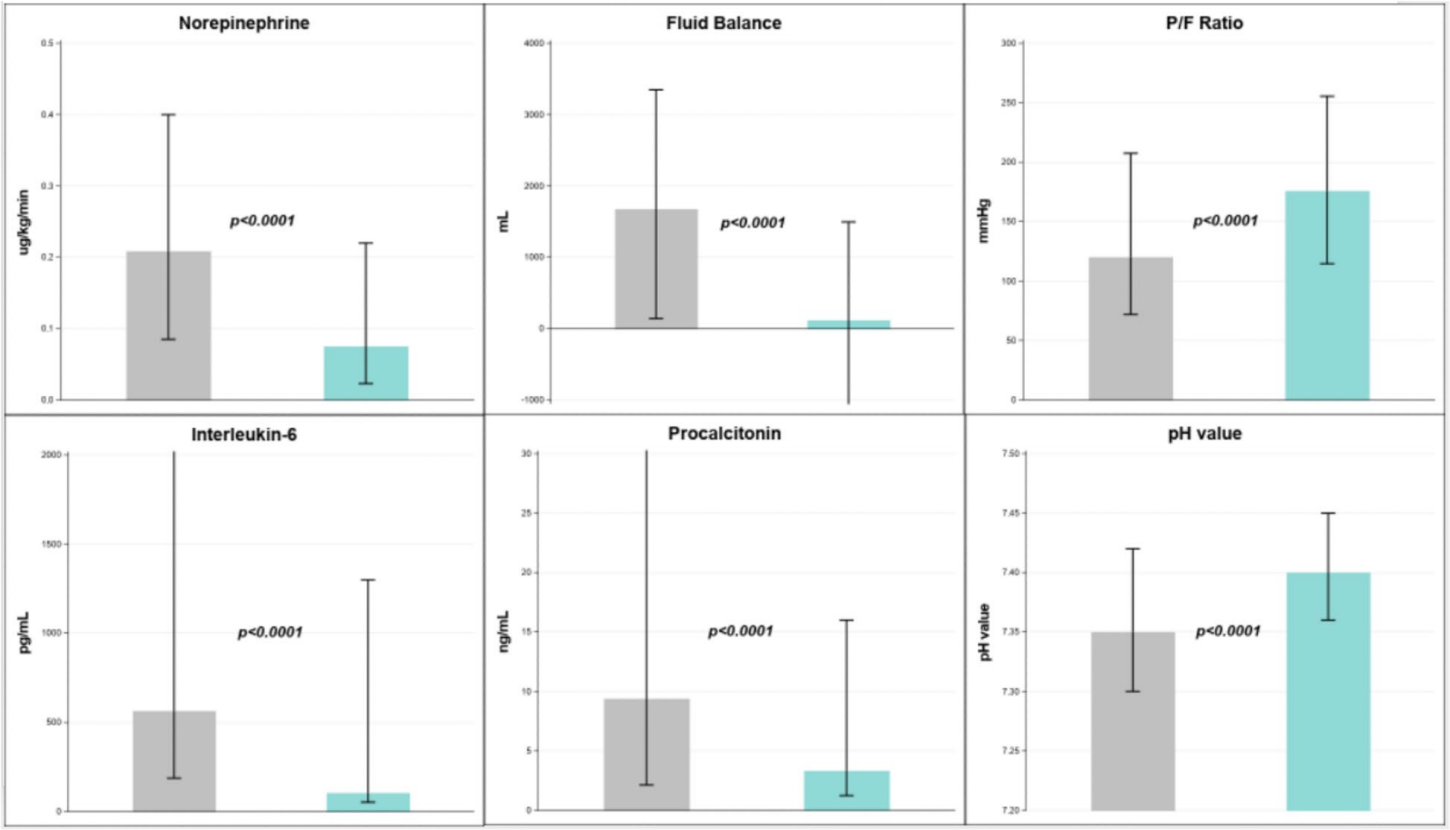
Changes in norepinephrine, fluid balance, P/F ratio, interleukin-6, and pH in 24 h periods before (grey) versus after CytoSorb® treatment (blue), data are presented as median and interquartile range. Legend: P/F ratio—ratio of partial pressure of oxygen in arterial blood to the fraction of inspiratory oxygen concentration

Secondary outcomes including 24-h fluid balance, norepinephrine requirements (NE), and P/F ratio all significantly improved post-CS treatment (*p* < 0.0001 for all, Fig. 2). Significant improvements were also noted post-treatment for MAP/NE index (287 [157, 635] to 681 [302, 1400] mmHg·kg·min/µg, *p* < 0.0001). Furthermore, change in fluid balance was also significant in patients without KRT from +1383 [0, 3700] to −227 [−1390, 497] mL (*p* = 0.034). SOFA score improved from 12 [9, 15] to 11 [8, 14] over the course of CS treatment in the whole cohort (*p* = 0.001), and equally from 12 [10, 15] to 11 [9, 14] in patients with septic shock (*p* = 0.003). Figure 2 summarizes the most important findings before versus after CS treatment.

### Safety

Platelet counts decreased during CS treatment (123 [76, 185] to 72 [42, 118] × 10^9^/L, *p* < 0.0001), an effect primarily driven by platelet count reductions in the septic cohort (122 [86, 182] to 65 [36, 121] × 10⁹/L, *p* < 0.0001), while platelets did not significantly decrease in patients with cardiogenic shock (102 [60, 164] to 69 [39, 107] × 10^9^/L, *p* = 0.137) or liver failure (85 [42, 149] to 66 [43, 99] × 10^9^/L, *p* = 0.322). Thirty-one percent of overall patients (*n* = 93) received a platelet transfusion concomitantly with CS therapy. No major bleeding events were reported. Median albumin levels did not change significantly during treatment (2.6 [2.3, 3.1] g/dL pre- versus 2.5 [2.3, 3.0] post-CS (*p* = 0.112)), however 43.7% of patients received albumin substitution. No serious adverse device effects or device deficiencies had been reported at the time this interim analysis was performed.

## Discussion

In this interim analysis of 300 patients, CS therapy was applied across diverse critical care indications. Observed improvements in hemodynamics, oxygenation and fluid balance, along with lower-than-predicted ICU mortality, may suggest potential benefit, although, in the absence of a control group, these findings should be interpreted cautiously.

These results are consistent with results from previous interim analyses [12]. Notably, the broad range of indications highlights its increasing role beyond septic shock, with emerging applications particularly in liver failure [13, 14] and rhabdomyolysis [10]. Further research is needed to clarify its role in these different contexts.

The mean number of adsorbers used reveals a high degree of variability in clinical practice, with 28% of patients receiving four or more devices. This underscores one of the major values of the registry, which enables a more comprehensive understanding of how clinicians indicate and apply CS therapy in real-world settings. As enrollment numbers in the COSMOS Registry continue to increase, dedicated subgroup analyses may help clarify how treatment intensity (e.g., number of adsorbers or exchange intervals) relates to clinical outcomes in different patient groups [9, 15]. Moreover, such analyses may identify specific subgroups that derive the greatest clinical benefit from CS therapy.

Patients included in this analysis had a median APACHE II score of 24 and a median SOFA score of 12, reflecting a high degree of critical illness severity. While these scores have historically been associated with high mortality risks in the populations from which they were derived, their ability to provide precise mortality estimates in contemporary ICU cohorts is limited, as advances in critical care over recent decades have substantially improved outcomes. A more recent study focusing on adults with suspected infection predicted for a SOFA of 12 an in-hospital mortality of around 50% [16]. In the current analysis, ICU mortality was 33.1% (32.9% in septic shock) despite high baseline disease severity. These findings are further supported by a recent systematic review and meta-analysis in septic shock patients, which showed that CS use significantly reduced both in-hospital and 28–30-day mortality in 744 critically ill patients [17]. While the design of this study does not allow for causal conclusions, these results may suggest a potential benefit of CS therapy in improving outcomes in critically ill patients, especially when used in appropriately selected patients and with adequate treatment regimens.

The observed median ICU stay of 22 days is notable in the context of healthcare resource utilization and cost. The potential clinical benefits—such as hemodynamic stabilization, improved oxygenation, and fluid balance—may translate into shorter ICU stays and reduced resource utilization if validated in larger controlled studies. Further cost-effectiveness analyses are warranted to determine its economic impact, especially in resource-intensive settings [18].

Norepinephrine requirements decreased significantly over the course of CS treatment resulting in hemodynamic stabilization, an observation that is consistent with prior evidence [19]. While the underlying pathophysiologic mechanism may be linked to improved vascular function and a reduction in the inflammatory burden [20], observed decreased vasopressor requirements with CS therapy may contribute to minimizing organ hypoperfusion and ischemia [21, 22]. Importantly, hemodynamic improvement was not achieved at the cost of additional fluid overload but was instead accompanied by significantly reduced need for fluid resuscitation which seems to be relevant also in the context of the observed improved oxygenation. These effects are clinically relevant, as intravenous fluid resuscitation can lead to dilutional coagulopathy, fluid overload, and the development of pathogenic edema in the lungs and other organs [23]. Reductions in lactate, creatinine, and fluid balance were also observed in patients not receiving KRT; however, these findings represent associations and cannot exclude the influence of other disease-specific therapies or supportive care. The observed improvements may reflect, at least in part, a potential endothelial protective effect of CS by removing circulating inflammatory factors known to impair endothelial function, contributing to enhanced vascular function and fluid homeostasis [20]. Furthermore, although a significant reduction in IL-6 levels was observed, this finding should be interpreted with caution. Although frequently used to assess the state of the inflammatory response and the response to therapeutic interventions, IL-6 has a very short half-life and levels can decrease rapidly even in the absence of extracorporeal therapies. Therefore, observed changes in IL-6 levels should be interpreted cautiously.

Finally, the current analysis reinforces the favorable safety profile of CS therapy, with no serious adverse device effects or deficiencies reported. The observed decrease in platelet counts, primarily in septic shock patients, should be interpreted cautiously, as thrombocytopenia is a common feature in sepsis [24]. It is however reassuring that despite the observed drops in platelet counts there were no bleeding events reported [25].

### Clinical implications

Timely removal of circulating harmful substances, such as overshooting cytokines [1] or myoglobin in rhabdomyolysis [5], is a critical goal in the management of critically ill patients. The hemoadsorption properties of CS enable efficient elimination of these and other inflammatory mediators, potentially contributing to the stabilization of the endothelial barrier [20] and attenuation of systemic hyperinflammation. COSMOS Registry data describe associations between CytoSorb use and beneficial changes in hemodynamics, fluid balance, and oxygenation. However, in the absence of a control group, these observations should be considered hypothesis-generating rather than evidence of treatment effect. Within this context, and from a clinical perspective, these associations may still be of practical relevance, particularly for physicians managing patients with refractory shock. Furthermore, improved fluid balance reduces risks associated with fluid overload, such as worsening respiratory distress reinforcing the potential benefit of CS therapy in managing patients with capillary leak and ARDS. Moreover, CS therapy was safely used in conjunction with renal replacement therapy, ECMO, or standalone hemoperfusion, underlining its versatility. Patients eligible for CS therapy should preferably be treated within 24 h of ICU admission. For decision-makers, these findings offer a rationale for further integration of CS therapy into standard care pathways while supporting ongoing research to maximize its clinical utility.

While these results are promising, further investigations are necessary, particularly controlled trials in dedicated indications that compare outcomes with the addition of CS therapy to established treatment standards. Such studies will help refine clinical indications, optimize treatment protocols, and further establish the role of CS in improving outcomes for critically ill patients. Importantly, observations from this registry may serve as a valuable source for hypothesis generation, guiding the design and focus of future clinical trials.

### Limitations

The major limitation of this registry analysis is the absence of a control group, which prevents attribution of observed changes to CytoSorb therapy and does not allow separation of potential treatment effects from concurrent disease-specific therapies, organ support, or natural disease course. As such, the findings should be interpreted with caution as suggestive rather than conclusive evidence. A second limitation is that at the time of this interim analysis, some patient datasets were incomplete, leading to a reduced sample size for certain analyses. Another limitation relates to the interpretation of mortality in relation to APACHE II and SOFA scores, which were developed decades ago and may overestimate expected mortality in contemporary ICU populations. Accordingly, these scores were used in this study to describe baseline disease severity rather than as accurate predictors of mortality. Furthermore, another limitation is the lack of standardized use of CS therapy across participating centers. The registry encompasses a heterogeneous population in which CS is applied for multiple indications. The therapeutic approach (treatment initiation, duration, and dosing strategies) may therefore differ according to the underlying indication and was left to the discretion of the treating physicians. In addition, there was no standardized measurement of predefined parameters, which may have introduced heterogeneity and limited comparability of outcomes across sites.

## Conclusions

This interim analysis of the international, real-world COSMOS Registry reports significant improvements in key therapeutic targets that might be associated with the use of CS as part of standard clinical practice across multiple critical care indications. Specifically, integration of CS was observed to be associated with hemodynamic stabilization as well as improved fluid balance and oxygenation resulting in observed ICU mortality that was lower than mortality estimates historically associated with established risk scores. Further controlled studies are required to determine whether the beneficial associations reported here represent actual treatment benefits of CytoSorb therapy.

## Data Availability

The data are available upon reasonable written request and with written permission of CytoSorbents Corporation. Data Management Plan and Informed Consent Forms are available upon request.

## Abbreviations

90-day FU: 90-Day follow-up
ACLF: Acute-on-chronic liver failure
ALF: Acute liver failure
APACHE: Acute Physiology and Chronic Health Evaluation
ARDS: Acute respiratory distress syndrome
BMI: Body mass index
CRP: C-reactive protein
CRS: Cytokine release syndrome
CS: CytoSorb®
DD: Device deficiencies
ECLS: Extracorporeal life support system
ECMO: Extracorporeal membrane oxygenation
FU: Follow-up
GCP: Good Clinical Practice
HLH: Hemophagocytic lymphohistiocytosis
ICF: Informed consent form
ICH: International Council on Harmonization
ICU: Intensive care unit
IEC: Independent Ethics Committee
IL-6: Interleukin-6
IQR: Interquartile range
IRB: Institutional Review Board
ISO: International Organization for Standardization
KRT: Kidney replacement therapy
MAP: Mean arterial pressure
NE: Norepinephrine
P/F ratio: Ratio of partial pressure of oxygen in arterial blood to the fraction of inspiratory oxygen concentration
RWE: Real-world evidence
SD: Standard deviation
SLEDD: Sustained low-efficiency daily dialysis
SOFA: Sequential Organ Failure Assessment
